# Changes in Salivary Amylase and Glucose in Diabetes: A Scoping Review

**DOI:** 10.3390/diagnostics11030453

**Published:** 2021-03-06

**Authors:** Pilar Pérez-Ros, Emmanuel Navarro-Flores, Ivan Julián-Rochina, Francisco Miguel Martínez-Arnau, Omar Cauli

**Affiliations:** 1 Frailty and Cognitive Impairment Research Group (FROG), University of Valencia, 46010 Valencia, Spain; maria.p.perez-ros@uv.es (P.P.-R.); emmanuel.navarro@uv.es (E.N.-F.); ivan.julian@uv.es (I.J.-R.); francisco.m.martinez@uv.es (F.M.M.-A.); 2 Nursing Department, University of Valencia, 46010 Valencia, Spain; 3 Department of Physiotherapy, University of Valencia, 46010 Valencia, Spain

**Keywords:** diabetes mellitus, salivary biomarker, glycated hemoglobin, glucose, amylase

## Abstract

Background and Objective: Diabetes mellitus (DM) is a common long-term disease which can be related with salivary amylase levels. DM has recently been associated with salivary amylase diagnostics that could further impair diagnoses in the diabetic population, as well as being an interesting alternative to traditional methods of determine glucose levels. The main advantage of this method is related to the fact that it is a fast diagnostic method. The DM population experiences changes to their metabolism which affects their salivary parameters, making this an alternative procedure for diagnosis and follow-up of the illness due to the non-invasive nature of salivary analyzes. The objective of this review is to summarize the evidence regarding the changes in salivary amylase and glucose levels, and their relationship with blood markers of glycemic control used in clinical settings such as blood glucose and glycated hemoglobin. The differences in salivary amylase levels depending on the method of saliva collection under fasting or non-fasting conditions. The changes in salivary amylase depends on the type of diabetes, the type of insulin treatment or the quality of glycemic control. Conclusions: Salivary amylase concentration is increased in diabetic patients in most of the studies and salivary glucose concentration in all studies in both fasting and non-fasting (post-prandial) conditions. Salivary amylase and glucose concentration represent potential non-invasive biomarkers to evaluate glycemic control and clinical management of diabetic patients, although it is necessary to evaluate the influence of potential modulating factors such as age, duration diseases, sex and the effects of pharmacological treatments in these outcomes which remained to be elucidated.

## 1. Introduction

Diabetes mellitus (DM) is a metabolic disease with a high prevalence worldwide, so it is an important global public health problem. Estimates suggest that 425 million people will have diabetes by 2025, which represents about 10% of the world’s habitants, and 90% of the diabetic population suffer from type 2 diabetes [1]. The acute complications of diabetes and its chronic complications, such as nephropathy, retinopathy, cardio-vascular diseases or diabetic foot, have been associated with hospitalizations and may be a cause of mortality [2,3].

The gold standard for measuring glycemic levels has traditionally been blood analysis of glucose and glycated hemoglobin by venous puncture and capillary venous puncture at home and subsequent use of glucometers. However the collection and analysis of blood test require an invasive approach and time to obtain the results. Point-of-care testing (POC), that is, the analysis of patients’ specimens outside the clinical laboratory, near or at the site of patient care, and usually performed by clinical staff without laboratory training, has recently been proposed as a rapid tool which is accessible for the patient and can be acted upon immediately. The key factor is the concept that clinical decision making may be delayed when samples are sent to the clinical laboratory [4]. This preventive action may cause unnecessary anxiety, especially in young populations [5] and people with neuropsychiatric disorders [6,7]. For these reasons, non-invasive procedures can be an alternative method for measuring glucose levels, which limits the possibility of stress-induced hyperglycemic states.

Salivary markers are non-invasive diagnosis tools that can overcome these limitations, and they can help clinical decisions at POC in diabetic patients as is the case with recently proposed salivary biomarkers in other pathologies. Salivary glucose is present in concentrations of 0.5 to 1 mg/dL, this increases mainly after the ingestion of food and beverages, as well as depending on the concentration of glucose in the blood. Prior research has shown good correlations between salivary glucose (stimulated and unstimulated conditions) levels using different techniques and glucose levels in blood [8]. Glycated proteins such as HbA1C can be compared with salivary markers, such as serum cortisol levels, salivary cortisol, plasma and prolactin levels [9], other putative diabetic markers [10], and the enzyme representing the first glycemic controlling enzyme in food digestion (i.e., salivary amylase) [11]. Moreover, fast blood glucose and salivary glucose test marks have been correlated significantly in patients with DM [12,13] and there is, in turn, a positive correlation between fast salivary glucose testing and HbA1c [12,14] and other salivary markers, for example, fructosamine glycated protein showed a significant correlation with HbA1c and blood glucose [15].

However the predictive value of the salivary glucose test can be modified due to bacterial flora in the mouth [15], hydration and certain drugs [13]. For this reason, this diagnosis method should be considered carefully and other salivary biomarkers could be more valid alternatives than glucose determination in saliva [16]. Blood glucose levels after starch intake are influenced by genetically determined differences in salivary amylase, an enzyme that breaks down dietary starches. In particular, the activity of higher salivary amylase is related to lower levels of blood glucose [17]. In fact, individuals with high concentrations of salivary amylase had significantly lower postprandial blood glucose responses following starch ingestion compared to individuals with low amount of the enzyme, this difference being apparently mediated by the increased plasma insulin concentrations in those individuals with high levels of the enzyme [17]. Nevertheless, both groups had similar plasma glucose and insulin responses following glucose ingestion. Thus, it is unlikely that group differences were due to innate differences either in their ability to produce insulin or in their capacity for insulin-mediated glucose disposal. Interestingly, the activity of salivary amylase has been associated with stress that increases it by stimulating the sympathetic autonomic nervous system, and as such it is considered a widely accepted marker of sympathetic activity in the body. Salivary amylase levels have been proposed as biological markers closely related to perceived stress in different physiological and pathological situations [18,19,20]. The measurement of salivary amylase is; therefore, an interesting useful marker for evaluating glycemic control in different pathological situations accompanied by an increase in the activation of the sympathetic system. In addition, which can; therefore, alter glycemic control and act as a marker of these stress-mediated changes in patients with diabetes.

The aim of this scoping review was to systemically evaluate the current evidence on employing salivary amylase and its associations with glycemic status in saliva in diabetic patients. A comparative analysis of salivary amylase concentration and activity was also performed for common blood glycemic parameters used in diabetes patients in clinical settings, such as blood glucose and HbA1c concentration.

## 2. Materials and Methods

We analyzed all original articles available in the most widely used scientific databases (e.g., in PubMed/Medline and Scopus), published until October 2020, with no date limitations and fulfilling the following inclusion criteria: (1) Full text in English, Spanish or Portuguese; (2) primary articles only; and (3) measurement of amylase levels in saliva; (4) diabetic patients. When determining the articles to include, we analyzed the title and abstract, and the full text for articles that fulfilled the inclusion criteria. Finally, the reference lists of all relevant articles were manually cross-referenced to identify additional articles. The search terms employed were “diabetes” AND “saliv*” AND “glucose” OR “amylase”).

Each article was evaluated by two independent reviewers, and any discrepancy was resolved by a third reviewer. Each reviewer evaluated the main characteristics of the studies described, indicating whether these fulfilled the eligibility criteria.

### Data Extraction

As a consequence of the large number of references to studies found in the database search, an Excel^®^ sheet was designed to facilitate the selection process, acting as a data collection form in which the codification of the items (criteria) to evaluate were clearly identified

## 3. Results

### 3.1. Summary of Identified Studies

**Figure 1:**
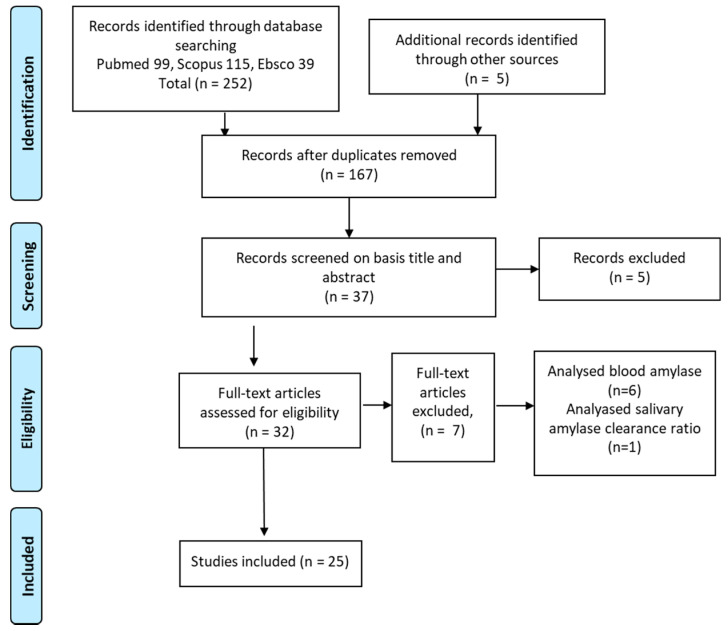
Preferred reporting items for systematic reviews and meta-analyzes (PRISMA) workflow for literature searches.

A total of 167 studies were found by searching in databases. After eliminating duplicates, 32 were analyzed to prepare the scoping review (Figure 1). After reading the full texts, seven of the studies were not analyzed due to failing to meet the inclusion criteria; six of them analyzed blood amylase [21,22,23,24,25,26], one of them studied the differential clearance of isoamylases [27]. Five researchers independently summarized the results extracted from these articles.

### 3.2. Main Characteristics of the Studies’ Subjects

Twenty-five of the included studies obtained the saliva sample directly from the oral fluid, and the remaining one [28] quantified the data by obtaining a biopsy specimen of the parotid gland. All the studies compared diabetic patients with healthy controls, except one longitudinal study [29] that analyzed a sample of diabetics at two points in time in different diabetic controls. In addition, several studies differentiated between controlled and uncontrolled diabetics within the diabetic group [30,31,32,33], or according to the presence of obesity [34] or according to the presence of neuropathy [35]. Table 1 shows the baseline characteristics of the 24 studies included. Most studies include adults with Type 2 diabetes (T2D) [11,30,34,36,37,38,39] or both types (T1D and T2D) [28,29,31,33,40,41], and in some studies, the participants were classified not according to the type of diabetes (i.e., type I or II), but instead based on their current insulin treatment (i.e., as non-insulin-dependent diabetes (NIDD) [42,43,44,45], insulin-dependent diabetes (IDD) [46] or both [47]). Finally, López et al. [48] and Hirtz [49] included T1D in children. Only three studies did not specify the type of diabetes [10,32,35].

### 3.3. Saliva and Blood Sampling

The saliva samples were obtained under fasted conditions in the morning before breakfast in seven studies [33,34,36,44,47,48,50] and from 1 to 2 h after a meal in thirteen studies [28,30,34,35,36,37,38,39,42,47,48,49,50]. In addition, most studies obtained the saliva sample without stimulation, while others obtained it after stimulation with paraffin [29,46,49] or citric acid [35]. Some studies also analyzed both unstimulated and stimulated individuals [31,37,42].

Some studies also collected blood samples under fasted conditions [33,40,43], and non-fasting/postprandial conditions [29,36,37,42] or both [11,44]. In addition, some of the samples were from veins [11,33] and from capillaries [36,43].

#### 3.3.1. Salivary Flow Rate in DM

The flow rate was analyzed in some studies, and only showed significant differences between the groups in unstimulated saliva samples in children with T1D [48], being lower in diabetic patients compared to the control group, although the increase falls within the normal range. On the other hand, in stimulated saliva samples, Ben-Aryeh et al. [37], Choukaife et al. [42] and Prathiba et al. [50] found significant differences in T2D, with lower rates in the diabetic groups. Newrik et al. [35] found the most significant differences between neuropathic individuals and controls (0.06 vs. 0.53 mL/min), but no differences were observed between non-neuropathic diabetic patients and non-diabetic individuals.

#### 3.3.2. Salivary Amylase Levels

The concentration of salivary amylase has been determined mainly by two techniques, that is, commercially available enzyme-linked immunosorbent assay (ELISA) based on a rapid immunochemical reaction test [30,36,41] and both amylase content and activity by biochemical assays based on colorimetric reactions employing chromogenic starch substrates [10,11,27,28,29,30,32,35,36,37,38,39,40,41,43,47,50]. Among enzymatic methods, the Phadebas^®^ method [51,52] is particularly easy to perform, shows high accuracy and is commercially available. Phadebas is a synthetic biochemical substrate used for both qualitative and quantitative assessment of the α-amylase enzyme. Its active component is DSM-P (degradable starch microspheres), in which a blue dye has been chemically bound. When the substrate is digested by the amylase enzyme, it releases that blue dye at a rate proportional to the quantity of the enzyme present. Amylase content can also quantified by immunocytochemistry technique in parotid gland tissue [28]. Finally, two studies [38,49] applied label-free differential protein expression analysis using mass spectrometry. Some studies analyzed differences in salivary amylase concentration by sex and age, and none of them found any differences and correlations by age [30,31,36,41,44,46,48].

In the unstimulated saliva samples, the amylase levels were statistically significantly higher in diabetic patients in ten studies [10,11,30,32,37,39,40,41,47] and also in the study by Piras et al. [28] performed in parotid gland tissue. The increase in amylase concentration was generally observed in both the fasting [34,36,48] and non-fasting samples [10,11,30,33,40,41]. In contrast, four studies [31,35,39,46] reported significantly lower levels in diabetic patients than in controls; three of them under non-fasting conditions [31,39,45] and only one in a fasting sample [50]. Among the most recent techniques to analyze protein expression in biological samples, proteomics provides high accuracy and sensitivity of proteome analysis; the hybrid platforms of multidimensional separations and mass spectrometry have provided the most powerful solution. Multidimensional separations provide enhanced peak capacity and reduce sample complexity, which enables mass spectrometry to analyze more proteins with high sensitivity [53]. The changes in amylase concentration in saliva samples in diabetic patients have been demonstrated by using two-dimensional gel electrophoresis coupled with matrix-assisted laser desorption/ionization time-of-flight mass spectrometry (MALDI-TOF/MS) [49] or multidimensional liquid chromatography/tandem mass spectrometry (2D-LC-MS/MS) [38]. Another three studies found no differences between the groups [32,44,47] (Table 2). In stimulated and non-fasting samples, only the study by Dodds et al. [43] also obtained higher levels for diabetic patients compared to the control group (Table 3).

#### 3.3.3. Salivary Glucose Levels and Hb1ac Levels

Salivary glucose levels were statistically higher in diabetic patients, ranging from 1.26 to 11 mg/dL, than in controls, ranging from 0.5 to 4.8 mg/dL. Significant differences were also observed between blood glucose levels, which ranged from 173 to 327 mg/dL in diabetics and 83 to 122 mg/dL in healthy controls. Hb1Ac was also higher in diabetic patients (ranges 7.22% to 17.3%) than in healthy controls. Analysis of the results concerning salivary glucose concentration showed that, in fasting conditions, there is a major increase in glucose concentration in the saliva of diabetic patients compared to its levels in blood samples. The magnitude of such an increase is two-fold in three studies [10,36,48] and in the majority of the studies the increase in salivary glucose concentration was by three-fold and more. The increase in salivary glucose is three times or more in diabetic patients than in controls, and it appears similar in fasting or in those studies in which salivary glucose concentration has been measured 1–2 h postprandial.

#### 3.3.4. Correlations between Salivary Amylase and Blood Glucose Levels

Only five studies correlate salivary amylase with salivary glucose concentration. The study of Panchbai et al. [31] showed a significant correlation in the uncontrolled group, whereby salivary amylase was lower in diabetic patients (although with very small statistical significance). On the other hand, in the study by Tiongco et al. [10], salivary amylase was higher in diabetics and they found a significant correlation between fasting blood glucose and salivary amylase (r = 0.226, *p* = 0.04) and also with salivary glucose (r = 0.416; *p* < 0.001). Three studies found no significant correlation [36,42,44].

In addition, there were correlations between salivary amylase and blood glucose levels in non-fasting samples, ranging from r = 0.138, *p* < 0.05 (43) to r = 0.226, *p* < 0.001 [10]. Indira et al. [39] and Kheirdman et al. [30] found no correlations.

As regards other correlation parameters, salivary amylase correlates with salivary total protein (r = 0.4842, *p* < 0.05) in the studies by Indira et al. [39], Panchbai et al. [31] and Ben-Aryeh et al. [37]. Lima-Aragao et al. [41] constructed a ROC curve to validate the salivary parameters that could be used for diagnostic testing. A test was considered positive in the event of alterations in glucose, total protein, urea, IgA and amylase concentrations. The sensitivity of the test was 88%, specificity was 90%, and the diagnostic accuracy was 89%. The salivary parameters of diabetic patients showed an AUC in salivary parameters of 0.99 for glucose, 0.98 for total protein, 0.95 for amylase, 0.84 for IgA, 0.81 for urea and 0.55 for calcium (all parameters *p* < 0.0001). Tiongco et al. [10] also showed an AUC in salivary glucose of 0.811 *p* < 0.001 and of 0.649 *p* < 0.05 in salivary amylase.

#### 3.3.5. Enzymatic Activity of Salivary Amylase in Diabetics

Artino et al. [47] measured salivary amylase activity (measured as the ratio to protein quantity and saliva volume to remove protein-related variations), which presented minimum levels in the morning and maximum levels in the afternoon. There were no significant differences between the groups. Reznick et al. [32] found no differences between the groups, but the amylase activity in the DM-uncontrolled group was substantial (by 122%, *p* = 0.07). Dodds et al. [43] attempted to determine whether alterations in glycemic control alter amylase activity. Paired saliva samples from subjects with blood glucose levels of at least 150 mg/dL who subsequently showed improved glycemic control (defined simply as a reduction in fasting blood glucose levels) were compared for amylase activity. A significant reduction in amylase activity and production (862 ± 94.3 before vs. 410.8 ± 76.5 after U/mL, *p* < 0.0001) occurred concomitantly with the fall in blood glucose levels. When the opposite situation was studied (i.e., patients showing increases in blood glucose (from levels ≥ 135 mg/dL to levels ≤ 170 mg/dL)), there was a non-significant increase in amylase activity (364 ± 51.7 before vs. 422 ± 74.3 after, U/mL *p* > 0.05).

#### 3.3.6. Correlation between Salivary Amylase and Diabetic Complications

Only Kheirdman et al. [30] analyzed the differences of salivary amylase in the presence of oral pathologies. The levels of salivary amylase were higher in oral candidiasis and erythematous candidiasis, but no other correlations with salivary IgA and periodontal disease were found.

Two studies [37,42] analyzed the presence of diabetic complications as clinical characteristics of sample. The prevalence of those complications was from 28.5% to 57.8% for skin problems, from 5.7% to 6.67% for nephropathy, from 24.4% to 25.7% for retinopathy, from 20% to 31.1% for neuropathy, and 8.5% for peripheral vascular disease. These studies did not analyze salivary amylase according to the prevalence of these complications.

## 4. Discussion

There has been increasing interest in salivary biomarkers in recent years. The main justification for their use is their ability to monitor how and when a disease starts and how it progresses, and to observe the outcome of treatment in promoting health and well-being. To that end, there must be specific biomarkers associated with the state of health or disease, which can be detected and monitored in a non-invasive way, and technologies that discriminate these biomarkers are required [54]. Salivary biomarkers meet the second requirement and, after analyzing research studies, the first and third are fulfilled. Salivary amylase plays an important role in the oral cavity. Both complex carbohydrates and simple carbohydrates changes into glucose [34]. Diabetes, due to its association with the autonomic system, modifies the quantity of saliva, the composition of amylase levels and other salivary biomarkers [50] related to catecholamine, and other substances such as cortisol. This scoping review endeavors to analyze the role of salivary amylase as a potential biomarker for diabetes mellitus, comparing the concentration of salivary amylase in diabetics (T1D, T2D, IDD and NID) with healthy controls or after an intervention to improve diabetic control. Although the first studies were published more than three decades ago, research on this subject has increased in the last ten years.

Salivary amylase starts the hydrolysis of starch in the mouth, and this process accounts for no more than 30% of the total hydrolysis of starch. Because salivary amylase is inactivated by an acidic pH, no significant hydrolysis of carbohydrates occurs in the stomach [55]. The acinar cells, which produce salivary amylase, are also innervated by sympathetic and parasympathetic pathways. Activation of the sympathetic nervous system increases amylase synthesis, which increases the concentration of amylase in saliva, and parasympathetic activity increases the saliva flow rate with little or no effect on amylase synthesis. Salivary amylase is related to the autonomic system and it is involved in in glycemic digestion, so it could be a good biomarker for assessment and follow-up DM, [56].

The heterogeneity of the studies analyzed in terms of type of diabetic population, together with the different ways results are presented by the authors, from how the saliva sample is collected to how the salivary amylase is expressed and what they really want to measure (concentration, secretion or activity), means that comparison of the results is difficult [57].

Most studies show higher levels of salivary amylase in DM patients in unstimulated samples. Diabetic patients have altered expression of amylase and cyclic adenosine monophosphate (cAMP) receptors in the parotid gland, and this could lead to changes in the production of salivary proteins, and particularly for salivary amylase [56]. In addition, there is an increase in the permeability of the basal membrane, which could allow a leakage of proteins in saliva through the salivary glands [10,40,50,58]. Only one study shows the same results in stimulated samples, and the others found no differences, which could be due to the mechanical stimulation of the saliva secretion changing the protein content of the saliva due to different content of the parotid and submandibular glands. Salivary flow is controlled by the autonomic nervous system, and mainly by the parasympathetic nervous system. The parasympathetic innervation of the parotid gland is caused by the glossopharyngeal nerve (cranial pair IX), via the optic ganglion. The facial nerve (cranial nerve VII) provides the parasympathetic innervation to the submandibular and sublingual glands, via the submandibular ganglion [54]. In passive sampling, only 20% of saliva will come from the parotid glands, which have more salivary amylase than the submaxillary and sublingual glands [59]. If they are stimulated, no differences in concentration are obtained and changes of between 25 to 40% can occur [57]. Other aspects that should be emphasized regarding the collection of saliva samples are that, in healthy people, salivary amylase has a particular diurnal profile, declining immediately after awakening and increasing constantly during the morning and afternoon [47,56]. Therefore, the collection of saliva samples should take place according to the same schedule (about 1 h after awakening) and the collection range should not be too long [31,37,46,60]. Lastly, the saliva collection method also interferes with the data obtained from salivary amylase. The use of cotton sponges could lead to salivary amylase measurement errors, with nearly complete salivary amylase retention when the cotton absorbs 0.25 mL of saliva. This means that the amount of saliva, which is related to the flow rate and/or duration of collection, will indirectly influence the salivary amylase levels. The drooling method or spitting method should; therefore, be used as a first step if there is no alteration of salivary flow, and absorbent products are required under conditions such as strenuous exercise or with patients with alterations in saliva secretion, such as xerostomia [57].

The differences in salivary amylase levels depending on the method of saliva collection under fasting conditions are uncertain, since differences with higher levels were observed under both fasting [34,36,48,60] and non-fasting conditions [11,30,32,41,61]. The heterogeneity of the results depending on the type of diabetes, the type of insulin treatment or control of the disease may also depend on whether the sample is collected under fasting or non-fasting conditions [62]. Conducting studies with uniform criteria would enable results to be unified for comparison.

Meanwhile, six studies [31,38,39,45,49,50] showed lower levels in diabetic patients than healthy controls. The authors attribute these levels to hormonal and metabolic changes in diabetic patients, such as microvascular complications and autonomic neuropathy, both of which may affect salivary secretions [35]. Hirtz [49], which uses mass spectrometry analysis, speculated whether the under-accumulation of α-amylase spots in diabetic patients could be related to changes in oral anti-inflammatory status. In addition, they also suggest that the diabetes would affect selectively only a part of α-amylase isoforms.

These apparent discrepancies could also be due to the saliva collection method, and other factors that could be involved in salivary amylase levels, such as years of evolution of DM [63], neurological comorbidity [56] such as Parkinson’s disease [64], and other pathologies that alter salivary flow such as gastro-esophageal reflux [65]. Other possible factors include the use of drugs that act on the parasympathetic system, such as pilocarpine, myorelaxants, anti-epileptic and anti-psychotic drugs; treatment that interferes with the action of acetylcholine, such as anticholinergics, antihistamines and cytostatic; and head and neck radiation therapy [54]. Therefore, all these aspects should be taken into account in the recruitment of subjects or as confounding factors in the analysis of data.

All the studies found higher levels of salivary glucose and blood glucose in diabetic patients, since this is a diagnostic criteria, but few studies analyzed their correlation with salivary amylase. When interpreting these results, the limitations on obtaining salivary amylase mentioned above could explain their variability. A positive correlation with blood parameters was observed for unstimulated and non-fasting samples [30,31,44]. Salivary amylase and blood glucose are positively correlated in studies with similar saliva sample collection characteristics. Salivary amylase also shows a good correlation for total salivary proteins [33,39] and with blood amylase [61]. It should be noted that, in these analyses, not all parameters present a good correlation between saliva and blood according to the studies above, in addition to variations in concentration depending on saliva flow in the case of polar or ionic compounds of high molecular weight transported by saliva or secreted by exocytosis [54].

Two studies reported correlations with several metabolites which could be used in the clinical setting as a diagnostic value in DM, and obtained the highest value for the area under the curve for salivary glucose, followed by salivary amylase [41,61].

Several authors analyzed enzymatic activity, but found no conclusive results, although its activity is increased in uncontrolled patients [32] and reduced in those who control their glucose levels [43]. More studies are necessary to better understand these aspects, since salivary amylase could play an important role in the follow-up of diabetic patients

Few studies analyzed the salivary amylase levels in the presence of DM complications. Salivary amylase secretion is directly related to the autonomic system, and the parasympathetic denervation of the parotid gland in diabetic neuropathy may follow a generalized distribution in autonomic neuropathy [35]. Two studies analyzed the prevalence of complications, but both obtained stimulated samples showing salivary amylase levels which were lower but not significant [42,66]. Only one showed increased salivary amylase in the presence of oral candidiasis [30], where saliva plays an important role in its immune function in both the control of bacteria and virus adherence [67].

Replacing blood tests with other samples such as saliva in order to perform a non-invasive process is becoming increasingly postulated for several pathologies, and it is particularly useful for those patients with neurocognitive disorders or children in which blood sampling, for instance, is very stressful. This is primarily because it is cheaper than determining blood levels, and it is a non-invasive procedure, and easy to store. It is also less infectious than blood, is easier to handle in diagnostic procedures and does not clot [54].

Although it is not possible to make clear recommendations about the use of salivary amylase measurements in diabetic patients for diagnostic purposes, the results of the scoping review suggest important technical and clinical issues for future studies in this research field. The recruitment of subjects should take into account the presence of comorbidities, years of suffering from DM and distinguish between T1D, T2DID and T2DNID. The possible drugs involved in obtaining saliva samples should also be assessed. The collection method should be unstimulated after 1 h awake and use a split or dropping method, if there are no problems such as xerostomia. The presence of complications related to the evolution of DM (neuropathy, nephropathy, retinopathy, dermatological alterations) must be considered in order to assess the prognostic levels of salivary amylase for DM assessment and to evaluate the effects of interventions aimed to improve glycemic status.

## 5. Conclusions

Salivary amylase content is increased in diabetic patients compared to non-diabetic individuals in most of the studies analyzed in this review. The increase in salivary amylase concentration was generally observed in samples collected in fasting and non-fasting (measured 1 to 2 h from meal intake) conditions. The majority of the studies reported an increase in salivary glucose concentration in individuals with diabetes by three-fold and more, suggesting similar biochemical alterations at the basis of the increase in these two biomarkers of glycemic index in saliva. The increase in salivary glucose appears consistent and replicated in saliva samples collected both after fasting and non-fasting conditions. However, a direct correlation analysis between the two salivary biomarkers (amylase and glucose) has been seldom investigated and the results are conflicting. No clear conclusions can be done regarding the association between salivary amylase changes in diabetes patients and glycemic control in blood and the presence of diabetic complications. Future studies are clearly necessary to address these issues for diagnostic purposes of putative salivary biomarkers.

## Figures and Tables

**Table 1:** Sociodemographic profile of subjects, type of diabetes mellitus (DM) and type of saliva and blood sampling.

Author, Year	n (Patients/Controls)	Type of DM	DM Patients (Mean ± SD; Median (Range)/% Men)	Controls (Mean ± SD;Median(Range)/% Men)	Saliva Sampling	Stimulation for Saliva Sampling	Concomitant Blood Sampling
Abd-Elraheem et al., 2017 [36]	20/20	T2D	47.6 ± 8.6/50	46.6 ± 8.4/50	Fasting, between 7 and 8 a.m.	Unstimulated	Postprandrial
Artino et al., 1998 [47]	17 ID, 14 NIDD/16	IDD and NIDD	39.7/47 ID, 56.4/50 DNID	43.8/50	Fasting before breakfast from 7:30 to 8:00 a.m.and fasting in the afternoon from 5:30 to 6:00 p.m. after at least 90 min of digestive rest	Unstimulated	NA
Aydin et al., 2007 [34]	20 O, 20 NO/22	T2D	47/55 O, 48/50 NO	49/45.5	Fasting, at 8 a.m. before breakfast	Unstimulated	NA
Ben-Aryeh et al., 1988 [37]	35/31	T2D	31.2 ± 7.4/57	29.0 ± 6.2/54.8	Non-fasting 1 h after meal from 8 to 11 a.m.	Two samples, the first unstimulated and the second stimulated with citric acid	At the same time as saliva sampling
Border et al., 2012 [38]	4/4	T2D and edentulous	Total Range (55–75)/25%	NA	From 9 a.m. to 12 p.m., after 120 min without oral stimulus	Unstimulated	NA
Choukaife el al., 2018 [42]	45/30	NIDD	30.7 ± 5.6/66.6	28.3 ± 5.4/66.6	Non-fasting 1 h after meal from 8 to 11 a.m.	Two samples, the first unstimulated and the second stimulated with citric acid	Non-fasting
Dodds et al., [43]	45/36	NIDD	50 ± 1.5/26.66	55.2 ± 1.7/36.11	Fasting (2 h)	Unstimulated	Fasting
Hirtz et al., 2006 [49]	8/8	T1D poorly controlled (HbA1C > 8% and 5 years at least of DM)	35.6 ± 9.9/75%	34.7 ± 8.2/NA	Non-fasting, 2 h after breakfast time	Stimulated by chewingon paraffin wax	NA
Indira et al., 2013 [39]	20/20	T2D	50.38 ± 6.57/50%		Non-fasting, 2 h after breakfast time	Unstimulated	NA
Kheirmand Parizi et al., 2019 [30]	30 U, 30 C/30	T2D	55.16 ± 2.2/33.3 U,50.76 ± 1.97/43.3 C	49 ± 1.4/46.6	Non-fasting 1 h after meal from 8 to 11 a.m.	Unstimulated	NA
Landgrota et al., 2016 [40]	60/60	T1D and T2D	52.32 ± 8.05/68.3	48.33 ± 7.30/53.3	Non-fasting, 2 h after breakfast time, from 9 to 11 a.m.	Unstimulated	Fasting
Lima-Aragão, 2016 [41]	88/39	T1D and T2D	52 ± 18/64.8	23 ± 6/43.6	Non-fasting	Unstimulated	NA
López et al., 2003 [48]	20/21	T1D	9.4 ± 3.9/45	8.3± 1.8/42.9	Fasting	Unstimulated	NA
Malathi et al., 2013 [11]	33/34	T2D NIDD	47.21 ± 7.82/50	46.44 ± 7.5/50	NA	NA	Fasting and postpandrial
Newrick et al., [35]	8NP,8NNP/8	NA	53 (32–78) NP/NA, 55 (35–83)NNP/NA	23 (18–30)	Non-fasting after a normal breakfast	Stimulated with citric acid	NA
Panchbhai et al., 2010 [31]	40 U, 40 C/40	T1D and T2D	48.50 ± 7.86/45 U49.50 ± 10.88/37.5 C	46.12 ± 10.25/40	Non-fasting after 1 h meal from 8 to 11 a.m.	Two samples, the first unstimulated and the second stimulated with citric acid	Na
Prathibba et al., 2013 [50]	30/30	T2D	48.14 (53.3)	44.44/46.6	Fasting between 7 and 8:30 a.m.	Unstimulated	NA
Piras et al., 2010 [28]	4 T1D, 5 T2D/11	T1D and T2D	Total Range (42–68)	-	Biopsia of parotid gland	-	NA
Reuterving et al., 1987 [29]	11/NA	T1D and T2D	28.7/72.7	NA	Fasting after 1 h meal before noon	Stimulated by parafilm	Fasting after 1 h eating
Reznick et al., 2006 [32]	11 U, 9 C/12	NA	15.1/50 U, 15/45.5 C	16.5/44.4	In the morning after 90 min without oral stimulus	Unstimulated	NA
Sathyapriya et al., 2016 [33]	60/25	T1D and T2D	56.5 ± 14.3/50	46.6 ± 18.2/50	Fasting from 8 to 11 a.m.	Unstimulated	Fasting, at 8 to 11 a.m.
Siddiqui et al., 2015 [44]	125/125	NIDD	46.91 ± 8.3/37.6	43.74 ± 7.54/34.4	Fasting at 8:00 a.m.	Unstimulated	Fasting and postpandrial
Tenovuo et al., 1986 [46]	35/35	IDD	30.4 (17–61)/68.57	Age and sex matched	Non-fasting after 1 h meal from 8 to 11 a.m.	Stimulated by parafilm	NA
Tiongco et al., 2019 [10]	25/55	NA	NA	NA	NA	Unstimulated	NA
Yavuzyilmaz et al., 1996 [45]	17/17	IDD and NIDD	54.23 ± 15.82/58.8	23.17 ± 3.26/41.4	Non-fasting after 1 h meal from 8 to 11 a.m.	Unstimulated	NA

NA: Not available, T1D: Type 1 diabetes, T2D: Type 2 diabetes, IDD: Insulin-dependent diabetes, NIDD: Non-insulin-dependent diabetes, O: Obese, NO: Non-obese and diabetic; U: Uncontrolled Diabetics, C: Controlled diabetics, A: Diabetics and albuminuria; MA: Diabetics and microalbuminuria.

**Table 2:** Unstimulated samples. Salivary amylase, flow rate, salivary glucose and blood glucose levels and correlations.

Author, Year	S-Amylase Units	S-Amylase Diabetics vs. Controls (Mean ± SD or Median (Range))	S-Amylase Diabetics vs. Controls	Flow rate (Mean ±SD or Median (Range)) (ml/min)	S-Glucose Diabetics vs. Controls (Mean ±SD or Median (Range)) (mg/dL)	Blood Glucose Diabetics vs. Controls (Mean ± SD or Median (Range)) (mg/dL)	Hb1AC Diabetics vs. Controls (Mean ± SD or Median (Range)) (%)	S-Amylase and S-Glucose Corre elations	S-Amylase and Blood Glucose Correlations	Other
Abd-Elraheem et al., 2017 [36]	U/L	2164.3 ± 578.2 vs. 885 ± 434 ***	Higher ***	NA	10.9 ± 10.11 vs. 4.8 ± 0.62 ***	PPBG 287 ± 34.65 vs. 122.2 ± 9.34 ***	7.22 ± 1.25 vs. 2.86 ± 0.56 ***	NS	NA	NS differences by sex and age in both groups
Artino et al., 1998 [47]	UI/L/g protein	Morning: 25,000 IDD, 175,000 NIDD vs. 190,000 ¥ Afternoon: 51,000 IDD, 390,000 NIDD vs. 40,500 ¥	NS	NA	NA	NA	NA	NA	NA	The increase in the salivary flow rate in the afternoon is accompanied by a decline in S-total protein concentration
Aydin et al., 2007 [34]	U/mL	628 ± 62 O, 612 ± 57 NO vs. 494 ± 44 O vs. Controls **, O vs. NO*, NO vs. Controls *	Higher *	0.97 ± 0.2 O, 1.09 ± 0.1 NO vs. 1.2 ± 0.3	3.9 ± 0.8 O, 3.8 ± 0.6 NO vs. 1.3 ± 0.3 O vs. C **, O vs. NO*, NO vs. C *	NA	NA	NA	NA	No differences between groups in total protein
Border et al., 2012 [38]	Spots	NA	Lower *	NA	NA	NA	NA	NA	NA	Reduced expression of salivary amylase in pooled samples from patients with diabetes compared to pooled control sample
Indira et al., 2013 [39]	U/mL	107.66 ± 28.60 vs. 154.96 ± 25.07 ***	Lower ***	NA	8.4 ± 4.59 vs. 1.65 ± 0.30 ***	282.25 ± 42.81vs 109.55 ± 11.19 ***	NA	r = −0.3328, NS	r = −0.3098, NS	Significant differences were found in S-total protein content, and correlations were found between S-total protein and S-amylase (r = 0.4842)* and S-glucose (r = −0.5181)*
Kheirmand Parizi et al., 2019 [30]	U/L	161,852 U vs. 95,793 C vs. 63,295 *** U vs. controls ** U vs. C	Higher ***	NA	NA	NA	r = −0.172 U, r = −0.166 C r = −0.096 Controls, NS	NA	r = −0.293 U, r = −0.222 C r = 0.096 Controls, NS	No correlation in S-amylase content by sex and age in both groups
Lodgrota et al., 2016 [40]		1671.42 ± 569.86 vs1397.59 ± 415.97	Higher **	NA	14.10 ± 6.99 vs. 5.87 ± 2.42 ***	211.50 ± 43.82 88.81 ± 11.29 ***	NA	NA	NA	-
Lima-Aragão, 2016 [41]	AU/dL	37 ± 0.1 vs. 37 ± 0.4 **	Higher **	NA	11 ± 2 vs. 3 ± 0.03 *	NA	NA	NA	NA	Nocorrelation between S-amylase by age
López et al., 2003 [48]	AU/dL	58.8 ± 37.4 vs. 35.5 ± 16.8 **	Higher **	0.2 ± 0.1 vs. 0.3 ± 0.1 ***	2.1 ± 1.6 vs. 1.0 ± 1.0 **	These parameters were inversely related to flow rate.	NA	NA	NA	NS differences in S-amylase by age S-glucose was poorly correlated with glycemia and with glycosylated hemoglobin; HbA1 S-amylase levels were lower than the levels in adults.
Malathi et al., 2013 [11]	U/L	2739.48 ± 1525.2 vs. 1740.38 ± 638.51 ***	Higher ***	NA	NA	173.88 ± 72.02 vs. 83.21 ± 9.84 ** PP 247.88 ± 86.37 vs. 141.62 ± 154.08 *	7.79 ± 1.15 vs. 5.15 ± 0.60 ***	NA	NA	The oral findings of 30 non-insulin-dependent diabetic patients revealed 7 patients with poor oral hygiene and halitosis and 12 patients showed periodontitis.. The other patients showed mild to moderate gingivitis.
Panchbhai et al., 2010 [31]	U/mL	108.48 ± 6.37 U vs. 100.83 ± 60.77 C vs. 146.72 ± 10.70* C vs. Controls	Lower*	Unst: 0.18 ± 0.12 U vs. 0.18 ± 0.14 C vs. 0.21 ± 0.20 St: 0.51 ± 0.27 U vs. 0.48 ± 0.29 C vs. 0.57 ± 0.35	8.09 ± 6.45 U vs. 7.65 ± 6.44 C vs. 1.89 ±1.44 ** U vs. Controls, C vs. Controls	NA	NA	With S-glucose * and S-total protein *** in U With S-total protein ** in C		No differences in S-amylase by sex between groups
Prathibba et al., 2013 [50]		19.20 ± 1.8 vs. 92.51 ± 13.74	Lower **	0.46 ± 0.02 vs. 0.67 ± 0.04 **	17.31 ± 2.05 vs. 4.33 ± 0.29 ***	NA	NA	NA	NA	-
Piras et al., año [28]	NA	10.27 ± 0.67 T1D vs. 2.83 ± 0.41 T2D vs. 3.27 ± 0.63 ** T1D vs. Controls	Higher **	NA	NA	NA	NA	NA	NA	-
Reznick et al., 2006 [32]	IU/L	988 (187–2596) U vs. 333 (18–3670) C vs. 466 (4–1968)	NS P = 0.078 U vs. Controls	NA	NA	NA	NA	NA	NA	-
Sathyapriya et al., 2016 [33]	U/mL	G2 (<100 mg/dL) 102.32 ± 67.61, G3 (100–150 mg/dL) 106.83 ± 60.77, G4 (150–250 mg/dL)108.48 ± 6.37, G5 (>250 mg/dL) 111.12 ± 11.94/96.72 ± 10.70*	Higher *	NA	G2 (<100 mg/dL) 7.30 ± 5.84, G3 (100–150 mg/dL) 7.64 ± 6.44, G4 (150–250 mg/dL) 8.09 ± 6.45, G5 (>250 mg/dL) 9.04 ± 7.17/5.91 ± 2.19 *	NA	NA	NA	NA	A correlation was found between S-amylase and S-total protein
Siddiqui et al., 2015 [44]	nKat/L	1.48 ± 1.15 vs. 1.24 ± 0.71	NS	NA	NA	NA	NA	NS	with PPBG (r = 0.138) *	No correlation in S-amylase by sex
Tiongco et al., 2019 [10]	U/L	930.8 ± 827.0 vs. 613.5 ± 667.3 *	Higher *	NA	12.6 ± 10.5 vs. 5.4 ± 8.7 **	174.5 ± 92.7 vs. 94.1 ± 17.4 **	S-glucose (r = 0.416) *** and FBG (r = 0.226) **	NA		AUC for DM diagnoses en S-glucose 0.811 ** and S-amylase 0.649 * Blood amylase levels: 71.7 ±21.7 vs. 92.2 ± 97.2, NS
Yavuzyilmaz et al., 1996 [45]	U/mL	124.2 ± 79.7 vs. 228.2 ± 185.5 *	Lower *	NA	NA	165 ± 51 vs. NA	NA	NA	NA	IDD 112.25 ± 76.37 NIDD 130.7 ± 82.2

¥ Estimated from graphic, NA: Not available, NS: Not significant, Significant at * *p* < 0.05 ** *p* < 0.001 *** *p* < 0.0001, S-amylase: Salivary amylase. S-glucose: Salivary glucose; S-total protein: Salivary total protein; S-ghrelin: Salivary ghrelin NA: Not available, PP: Post-prandial, PPBG: Postprandial blood glucose, FBG: Fasting blood glucose, T1D: Type 1 diabetes, T2D: Type 2 diabetes, IDD: Insulin-dependent diabetes, NIDD Non-insulin-dependent diabetes, O: Obese and diabetic, NO: Non obese and diabetic; U: Uncontrolled diabetics, C: Controlled diabetics, AUC: Area under the curve, G2: Blood sugar level < 100 mg/dL; G3: Blood sugar level 100–150 mg/dL, G4: Blood sugar level 150–250 mg/dL, G5: Blood sugar level > 250 mg/dL.

**Table 3:** Stimulated samples. Salivary amylase, Flow rate, Salivary glucose and blood glucose levels and correlations.

Author, year.	S-Amylase Units	S-Amylase Diabetics vs. Controls (Mean ± SD or Median (Range))	S-Amylase Diabetics vs. Controls	Flow rate (Mean ± SD or Median (Range)) (ml/min)	S-Glucose Diabetics vs. Controls (Mean ± SD or Median (Range)) (mg/dL)	Blood Glucose Diabetics vs. Controls (Mean ± SD or Median (Range)) (mg/dL)	Hb1AC Diabetics vs. Controls (Mean ± SD or Median (Range)) (%)	S-Amylase and S-Glucose Correlations	S-Amylase and Blood Glucose Correlations	Other
Ben-Aryeh et al., 1988 [37]	10^2^ IU/£	WR: 6026 ± 3753 vs. 6325 ± 4003 RP: 11,287 ± 3159 vs. 11,861 ± 4592 SP: 9930 ± 4089 vs. 11,200 ± 3140	NS	0.35 ± 0.24 vs. 0.48 ± 0.23 *	WR: 2.9 ± 5.8 vs. 1.5 ± 1.0 RP:3.2 ± 2.7 vs. 0.7 ± 0.6 *** SP: 1.9 ± 1.6 vs. 0.3 ± 0.3 ***	236 ± 66 vs. 80 ± 10 ***	NA	NA	NA	No differences in amylase activity between groups. Significant correlation between S-amylase and total protein in the control group.
Choukaife el al., 2018 [42]	10^2^ IU/£	WR: 5022 ± 2417 vs. 7590 ± 3652 RP:10,064 ± 4227 vs. 113,425 ± 66,457 SP:8697 ± 4125 vs. 12,465 ± 5920	NS	0,29 ± 0,17 vs. 0,58 ± 0,26 *	WR 3.48 ± 6.11 vs. 1.28 ± 0.88 RP 3.82 ± 2.90 vs. 0.58 ± 0.26 *** SP 2.27 ± 1.82 vs. 0.25 ± 0.1 ***	283 ± 71 vs. 68 ± 6.62 **	NA	NS	NA	No differences were found in Na, S-IgA Differences in proteins were found in WS and SP and differences in k were found in RPS, SP and WS
Hirtz et al., 2006 [49]	Spots	NA	Lower *	NA	NA	NA	NA	NA	NA	The spots were detected in nearly all subjects and showed an average five-fold under-accumulations in diabetic patients
Dodds et al., [43]	U/ml	537.0 ± 36.3 vs. 431.2 ± 30.08 *	Higher *	WR 0.41 ± 0.04 vs. 0.45 ± 0.05 SP 0.34 ± 0.03 vs. 0.35 ± 0.03	NA	198.6 ± 10.3 vs. 97.3 ± 3.3 ***	NA	NA	NA	Amylase activity before/after in the same group: SRBG 862 ± 94.3 vs410.8 ± 76.5 *** SIBG 364 ± 51.7 vs. 422 ± 74.3, NS
Newrick et al., [35]	IU/l	1144(514–5048) NNP vs. 488 (123–2443) NP vs. 727 (242–1400)	NS	0.55 (0.31–0.8) NNP, 0.15 (0.06–0.36) NP vs. 0.68 (0.53–0.85) ** NP vs. Controls	NA	261.32 (180–360) NNP vs. 216 (144–252) NPNA Controls	12 (7–19) NNP vs. 12 (9–14) NP	NA	NA	-
Reuterving et al., 1987 [29]	U/ml	One group, two moments: 0.33 ± 0.04 Fst vs. 0.55 ± 0.18 Snd	-	WR:0.05 ± 0.02 vs. 0.09 ± 0.02 RP:0.04 ± 0.012 vs. 0.07 ± 0.03 SP:0.62 ± 0.11 vs. 0.57 ± 0.07	WR:4.32 ± 0.72 vs. 1.26 ± 0.18 * RP:4.14 ± 1.26 vs. 1.62 ± 0.36 * SP:1.26 ± 0.36 vs. 0.36 ± 0.18 **	327.35± 158.54 vs. 105.21 ± 48.46	11.5 ± 1.86 vs. 7.92 ± 1.78 **	NA	NA	No difference in controlled or uncontrolled diabetics
Tenovuo et al., 1986 [46]	U/mL3	233 ± 154 vs. 277 ± 136	NS	1.47 0.63 vs. 1.62 ± 0.74	NA	NA		NA		No correlation for S-amylase by age. Diabetics have more Iga, IgG and peroxidase activity in saliva than controls

S-amylase: Salivary amylase. S-glucose: Salivary glucose WR: Whole resting; RP: Resting parotid; SP: Stimulated parotid; S-IgA: Salivary IgA ¥ Estimated from graphic, Significant at * *p* < 0.05 ** *p* < 0.001 *** *p* < 0.0001, NA: Not available, PPBG: Post prandial blood glucose, T1D: Type 1 diabetes, T2D: Type 2 diabetes, IDD: insulin-dependent diabetes, NIDD Non-insulin-dependent diabetes, NDD: New diagnosed diabetes, O: Obese, NO: Non-obese and diabetic; U: Uncontrolled diabetics, C: Controlled diabetics, A: Diabetics and albuminuria; MA: Diabetics and microalbuminuria, AUC: Area under the curve. Fst: First moment worse control, Snd: Second better control; SRBG: Subjects with reduced blood glucose, SIBG: Subjects with increased blood glucose.
